# Clinical Implication of Anti-Angiogenic Effect of Regorafenib in Metastatic Colorectal Cancer

**DOI:** 10.1371/journal.pone.0145004

**Published:** 2015-12-15

**Authors:** Yoojoo Lim, Sae-Won Han, Jeong Hee Yoon, Jeong Min Lee, Jung Min Lee, Jin Chul Paeng, Jae-Kyung Won, Gyeong Hoon Kang, Seung-Yong Jeong, Kyu Joo Park, Kyung-Hun Lee, Jee Hyun Kim, Tae-You Kim

**Affiliations:** 1 Department of Internal Medicine, Seoul National University Hospital, Seoul, Korea; 2 Cancer Research Institute, Seoul National University College of Medicine, Seoul, Korea; 3 Department of Radiology, Seoul National University Hospital, Seoul, Korea; 4 Department of Nuclear Medicine, Seoul National University Hospital, Seoul, Korea; 5 Department of Pathology, Seoul National University Hospital, Seoul, Korea; 6 Department of Surgery, Seoul National University Hospital, Seoul, Korea; 7 Department of Internal Medicine, Seoul National University Bundang Hospital, Seongnam-si, Geyonggi-do, Korea; 8 Department of Molecular Medicine & Biopharmaceutical Sciences, Graduate School of Convergence Science and Technology, Seoul National University, Seoul, Korea; University Campus Bio-Medico, ITALY

## Abstract

**Background:**

Regorafenib induces distinct radiological changes that represent its anti-angiogenic effect. However, clinical implication of the changes is unclear.

**Methods:**

Tumor attenuation as measured by Hounsfield units (HU) in contrast-enhanced computed tomography (CT) and cavitary changes of lung metastases were analyzed in association with treatment outcome of metastatic colorectal cancer patients (N = 80) treated with regorafenib in a prospective study.

**Results:**

141 lesions in 72 patients were analyzed with HU. After 2 cycles of regorafenib, 87.5% of patients showed decrease of HU (Median change -23.9%, range -61.5%–20.7%). Lesional attenuation change was modestly associated with metabolic changes of 18-fluoro-deoxyglucose positron emission tomography-CT (Pearson’s r = 0.37, *p* = 0.002). Among 53 patients with lung metastases, 17 (32.1%) developed cavitary changes. There were no differences in disease control rate, progression-free survival, or overall survival according to the radiological changes. At the time of progressive disease (PD) according to RECIST 1.1, HU was lower than baseline in 86.0% (43/50) and cavitary change of lung metastasis persisted without refilling in 84.6% (11/13).

**Conclusion:**

Regorafenib showed prominent anti-angiogenic effect in colorectal cancer, but the changes were not associated with treatment outcome. However, the anti-angiogenic effects persisted at the time of PD, which suggests that we may need to develop new treatment strategies.

## Introduction

Regorafenib is an oral multikinase inhibitor, which has anti-angiogenic and anti-tumor activity by inhibition of a number of angiogenic and oncogenic kinases [1]. It has shown clinical activity as a single agent in colorectal cancer and gastrointestinal stromal tumor (GIST) in phase 3 trials [2–4]. Regorafenib improved progression-free survival (PFS) and overall survival (OS) in previously treated refractory metastatic colorectal cancer [2, 4].

Progression-free survival data from the phase 3 trial of regorafenib in colorectal cancer suggest that the benefit of regorafenib may be limited to a subgroup of patients [2]. However, predictive biomarker for regorafenib has not been identified thus far [5, 6]. We are conducting a prospective exploratory study with an aim to discover predictive biomarker candidates of regorafenib in colorectal cancer using tumor tissue and serial blood samples.

During the course of regorafenib treatment in patients enrolled into the study, we observed distinct radiological changes after treatment with regorafenib. Tumor attenuation in contrast-enhanced computed tomography (CT) scans decreased in the majority of patients and cavitary change of lung metastasis was frequently observed. Considering the potent anti-angiogenic activity of regorafenib in preclinical xenograft models [1, 7–9], decrease in contrast enhancement may be a good pharmacodynamic surrogate. However, clinical implications of these anti-angiogenic radiological changes, especially its association with treatment outcome in regorafenib-treated patients, have not been comprehensively studied yet.

In the present study, we have analyzed the change of tumor attenuation measured by Hounsfield units (HU) after treatment and the occurrence of cavitary change in pre-existing lung metastasis in colorectal cancer patients treated with regorafenib in the prospective exploratory biomarker study.

## Materials and Methods

### Patients and Treatment

All patients included in the present analysis were a part of a main study entitled Identification of Predictive Biomarker of Regorafenib in Refractory Colorectal Cancer: A Prospective Explorative Study (NCT01996969). The key inclusion criteria of the main study are, age ≥20 years; pathologically proven metastatic adenocarcinoma of colon or rectum; failure of standard therapies, which must include fluoropyrimidine, oxaliplatin, and irinotecan, but not necessarily bevacizumab or cetuximab; measurable or non-measurable disease according to Response Evaluation Criteria in Solid Tumors (RECIST) criteria, version 1.1[10]; adequate tissue for gene sequencing; and ECOG performance status 0 or 1. Patients received 160 mg of regorafenib orally once daily, on days 1 to 21 of a planned 28-day cycle. Regorafenib was provided by Bayer Pharma AG (Berlin, Germany). Treatment was continued until disease progression or unacceptable toxicity. Response evaluation using contrast-enhanced CT was repeated every 2 cycles and tumor response was determined according to the RECIST 1.1. The protocol was later amended to analyze the radiological changes and to include fusion 18-fluoro-deoxyglucose (FDG) positron emission tomography (PET)-CT scans at baseline and at first response evaluation after 2 cycles. Patients with measurable lesions who had at least one response evaluation were included in the present imaging analysis.

All patients provided written informed consent before any study-specific procedures. The study protocol was approved by the Institutional Review Board (IRB) of Seoul National University Hospital [IRB number: 1307-144-507] and was conducted in accordance with the Declaration of Helsinki.

### Imaging Analysis

All patients underwent multiphasic abdominal CT consisting of precontrast, arterial, and portal venous phase images at multi-row detector CT scanners (Sensation 16, Siemens Healthcare, Forchheim, Germany) after eight hours of fasting according to the standard protocol of our institution. CT scans were performed using a standard kilovoltage (120 kVp) and an automatic tube-current modulation technique that modulates the tube current based on the patient’s geometry and anatomy. After scanning the precontrast image, contrast media (1.5 mg/kg, Ultravist 370, Bayer Schering Pharma, Berlin, Germany) was administered intravenously at a rate of 2.0–4.0 mL/sec using a power injector followed by a 30–40 mL saline flush. Arterial phase was obtained by using bolus tracking method and portal venous phase was obtained approximately 180 seconds after the beginning of contrast media administration. In addition to target lesion selection, size measurement, and response evaluation according to the RECIST 1.1,[10] we measured the CT attenuation coefficients of all target lesions and non-target lesions included in the correlation analysis with mSUV, in Hounsfield units (HU). HU measurement was performed by drawing free-hand region of interest as large as possible on portal venous phase. Mean HU of all target lesions excluding lung metastasis of each patient was calculated. HU was measured from the same target lesions in the follow-up CT. Lesional attenuation changes between baseline and follow-up CT scans after 2 cycles were calculated as follows: attenuation change (%) = (HU _follow-up CT_−HU _baseline CT_) / HU _baseline CT_ x 100. In analysis of tumor attenuation change per patient, mean HU of target lesions in baseline and follow-up CT were used. Lung lesions were excluded from the HU analysis because the air density in the lung metastasis yields a large negative HU value unrelated to the tumor enhancement.

In patients with lung metastasis, cavitary change occurring in any of the lung lesions was regarded as positive. Cavitary change included new appearance of visible air cavity in solid lung metastatic lesion and increase in cavity size with decrease in solid portion of pre-existing cavitary lung metastasis after treatment. Refilling of cavitation was defined by any increase in solid portion accompanied by decrease in air containing cavity. All CT images were independently reviewed by two experienced radiologists blinded to clinical data.

FDG PET-CT images were acquired according to our standard imaging protocol, using dedicated PET-CT scanners (Biograph 64 or mCT, Siemens Healthcare). Patients fasted for at least six hours before intravenous FDG (5.18 MBq/kg) injection and images were acquired 60 minutes after the injection. CT scan was performed first for attenuation correction and lesion localization, and emission scan was obtained from skull base to proximal thigh for one minute per bed position. PET images were reconstructed by an iterative algorithm and were reviewed by two experienced nuclear medicine physicians blinded to clinical data. On baseline and follow-up PET-CT images, the maximum standardized uptake values (mSUV) of the target lesions and all discernible lesions were measured using a dedicated analysis software package (*syngo*.via, Siemens Healthcare). The change of mSUV was calculated for each lesion and the correlation between HU change and mSUV change was analyzed. Measurable tumor lesions in addition to the target lesions were selected for the correlation analysis.

### Statistical Analysis

Categorical variables were compared using chi-square test or Fisher’s exact test as appropriate. Paired t-test was used to compare HU before and after treatment. Correlation between the changes of size, HU, and mSUV was assessed with Pearson’s correlation coefficient. To determine the cut-off point of HU change predicting any decrease in mSUV (change<0%) with the highest sensitivity and specificity, the receiver operating characteristic (ROC) curves were calculated and the Youden’s index was used OS and PFS were calculated from the first dose of regorafenib to death from any cause and progression of disease or death, respectively. Survival analysis was performed using the Kaplan-Meier method and comparisons were made with the log-rank test. Two-sided *p*-values of less than 0.05 were considered statistically significant. All statistical analyses were performed with IBM SPSS version 20.0 (IBM Corp., Armonk, NY).

## Results

### Patient Characteristics and Regorafenib Treatment

A total of 80 patients were included in the present analysis. Patient characteristics are summarized in Table 1. All patients have been previously treated with fluoropyrimidine, oxaliplatin, and irinotecan, while 11.3% and 13.3% of patients have received bevacizumab and cetuximab, respectively. More than half of the patients have been treated with three or more palliative chemotherapy regimens.

**Table 1:** Patient characteristics.

	Number of patients (%) (N = 80)
**Age, median (range)**	58 (26–77)
**Sex**	
Male	50 (62.5%)
Female	30 (37.5%)
**ECOG performance status**	
0	62 (77.5%)
1	18 (22.5%)
**Primary site of disease**	
Proximal colon	16 (20.0%)
Distal colon	33 (41.3%)
Rectum	31 (38.8%)
**Number of metastatic organs**	
1	17 (21.3%)
2	28 (35.0%)
≥ 3	35 (43.8%)
**Number of previous palliative chemotherapy regimens**	
≤ 2	33 (41.3%)
3	32 (40.0%)
≥ 4	15 (18.8%)
**Disease status**	
Initially metastatic	45 (56.3%)
Recurrent	35 (43.8%)
**Time from metastasis to enroll**	
Median months (range)	24 (6–106)
**Previous chemotherapy agents used**	
Fluoropyrimidine	80 (100%)
Oxaliplatin	80 (100%)
Irinotecan	80 (100%)
Bevacizumab	9 (11.3%)
Cetuximab (in 45 KRAS WT)	6 (13.3%)

**Fig 1:**
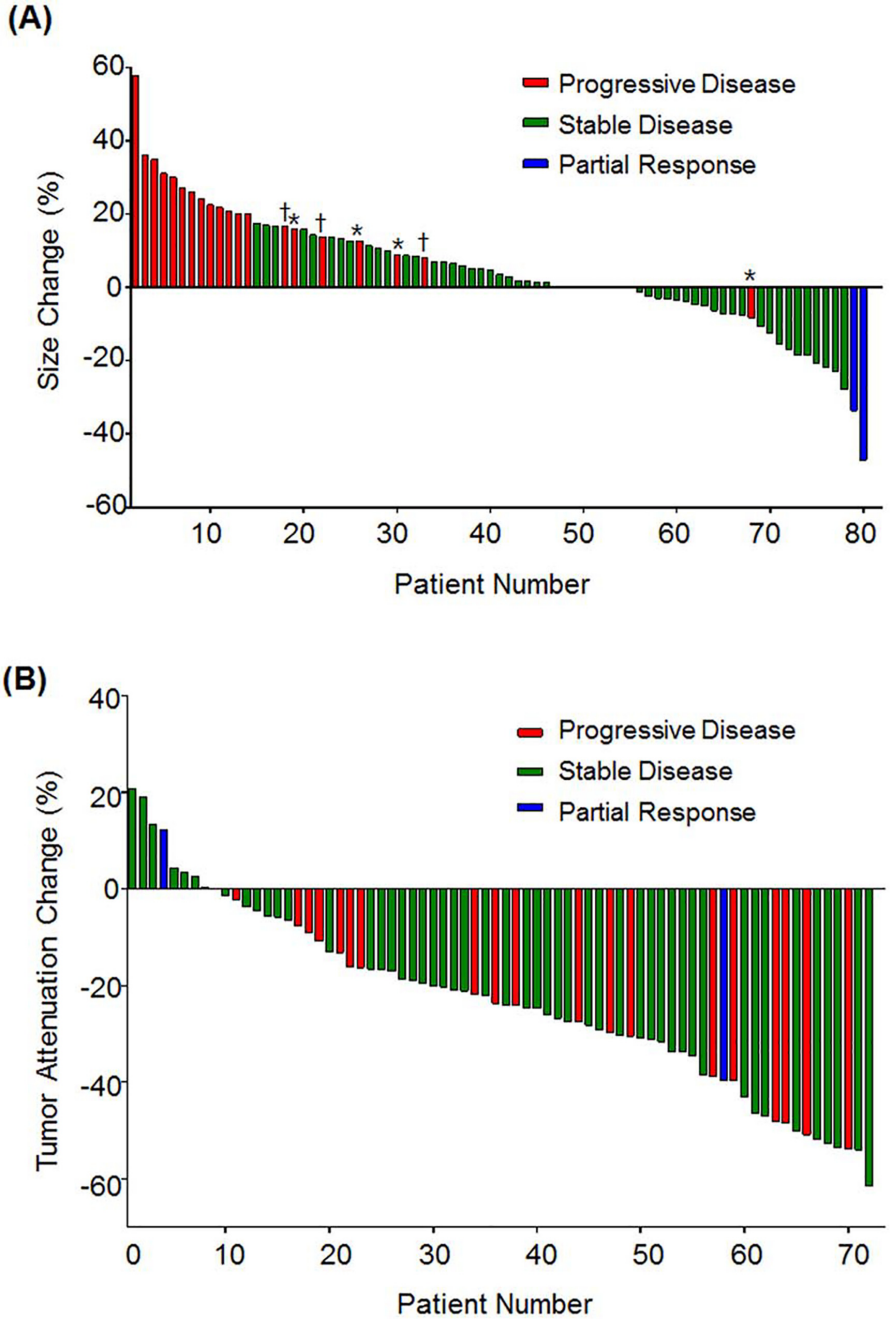
Waterfall plots of changes in tumor size and attenuation. (A) Size changes of target lesions from baseline. * Progression due to appearance of new lesion. † Progression of non-target lesion. (B) Tumor attenuation changes of target lesions from baseline after 2 cycles of treatment.

The best overall response of regorafenib according to RECIST 1.1 was partial response (PR) in two patients (2.5%), stable disease (SD) in 58 (72.5%), and progressive disease (PD) in 20 (25.0%). Of the 58 SD patients, 22 patients showed any reduction in the tumor size (Fig 1A). During a median follow-up duration of 9.0 months (range 3.2–12.7), 53 patients (66.3%) had disease progression and 31 deaths (38.8%) were observed which were all related to progression of cancer. Median PFS was 4.3 months (95% CI 3.0–5.5) and median OS 10.1 months (95% CI 7.7–12.4).

### Tumor Attenuation Change

A total of 141 lesions from 72 patients were analyzed for tumor attenuation with HU measurement (95 hepatic and 46 extrahepatic lesions). Median baseline tumor attenuation of the lesions analyzed was 61.8 HU (range 18.0–195.3). No significant difference in lesional attenuation was found between liver and extrahepatic metastasis (median 62.2 HU and 61.6 HU, respectively; *p* = 0.57).

**Fig 2:**
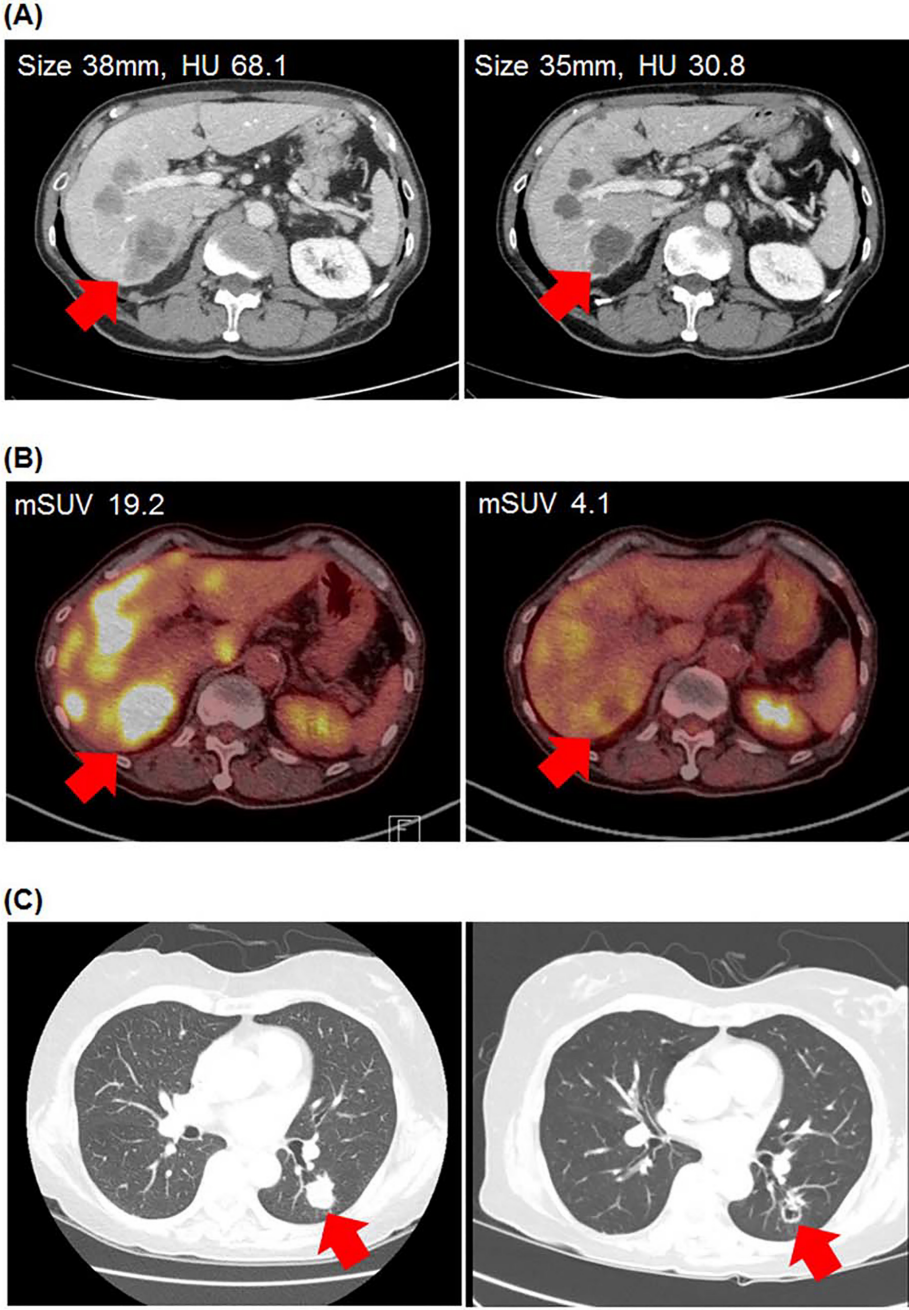
Representative images of anti-angiogenic changes of regorafenib. (A) Tumor size and attenuation in baseline CT (left) and follow-up CT after 2 cycles of regorafenib treatment (right). (B) PET-CT images of the same target lesion before (left) and after 2 cycles (right). (C) Cavitary change of lung metastasis. Images of baseline CT (left) and follow-up CT after 2 cycles (right).

**Fig 3:**
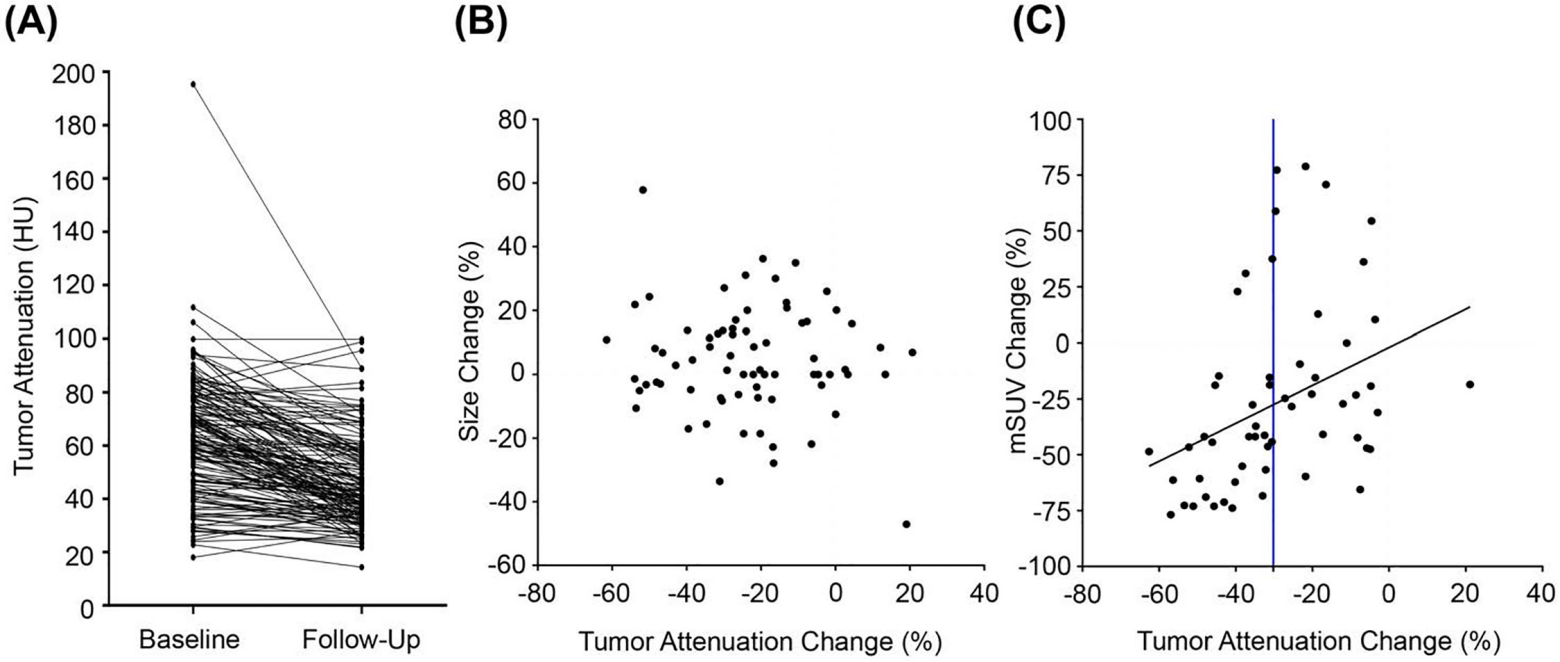
Tumor attenuation changes after regorafenib treatment. (A) Lesional attenuation of target lesions in baseline and follow-up CT scans. (B) Scatter plot of tumor attenuation change and size change from baseline. (C) Scatter plot of lesional attenuation change and mSUV change. Blue line indicates cut-off from ROC curve analysis.

After two cycles of regorafenib treatment, tumor attenuation in HU decreased in the majority of the lesions (Fig 2A). Decrease in tumor attenuation was observed in 83.0% of the lesions, whereas 17.0% of lesions showed increase (Fig 3A). Median lesional attenuation change was -24.0% (range -63.5–55.5). Liver metastatic lesions showed more prominent lesional attenuation change compared to extrahepatic metastatic lesions (median -28.6% *vs*. -18.7%, respectively; p<0.001).

Tumor attenuation change per patient ranged from -61.5% to 20.7%, with a median of -23.9%. 87.5% of patients showed tumor attenuation decrease after regorafenib treatment (Fig 1B). There was no correlation between the degree of tumor attenuation change and size change according to RECIST 1.1 (Pearson’s r = -0.11, *p* = 0.36, Fig 3B).

The correlation between lesional attenuation change and mSUV change after two cycles of treatment was analyzed in 64 matched lesions from 12 consecutive patients included after the amendment of protocol (Fig 2B, S1 File). Lesional attenuation change was modestly associated with mSUV change (Pearson’s r = 0.37, *p* = 0.002, Fig 3C). Using a cut-off (-30.52%) derived by ROC analysis, 93.5% of lesions with higher HU decrease (decrease>-30.52%) showed mSUV decrease from baseline, whereas 63.6% showed mSUV decrease in the other lesions (HU increase or decrease<-30.52%) (*p* = 0.004).

### Cavitary Change of Lung Metastasis

Cavitary change after treatment was analyzed in 53 patients with measurable or non-measurable lung metastasis. 35 patients had measurable lung metastasis (≥1cm), while the size of lung lesions in the other 18 patients ranged from five to nine millimeters. Eight patients had pre-existing cavitary lung metastasis. Among the 53 patients, 17 patients (32.1%) developed cavitary change (Fig 2C). New cavitation was observed in 15 patients with the smallest observed cavity diameter at 2mm. Six patients showed increase in pre-existing cavity size. The cavitary changes were noted at the first CT evaluation in all of the patients who developed cavitary changes. In the 35 patients with measurable lung metastasis, 13 patients developed cavitary change in the measurable lesion.

Subsequent refilling of cavitary change was observed in three out of 17 patients at the time of data cut-off.The refilling preceded the PD in two patients by two and four months each, while the other patient was still on treatment.

### Anti-Angiogenic Changes and Treatment Outcome

**Fig 4:**
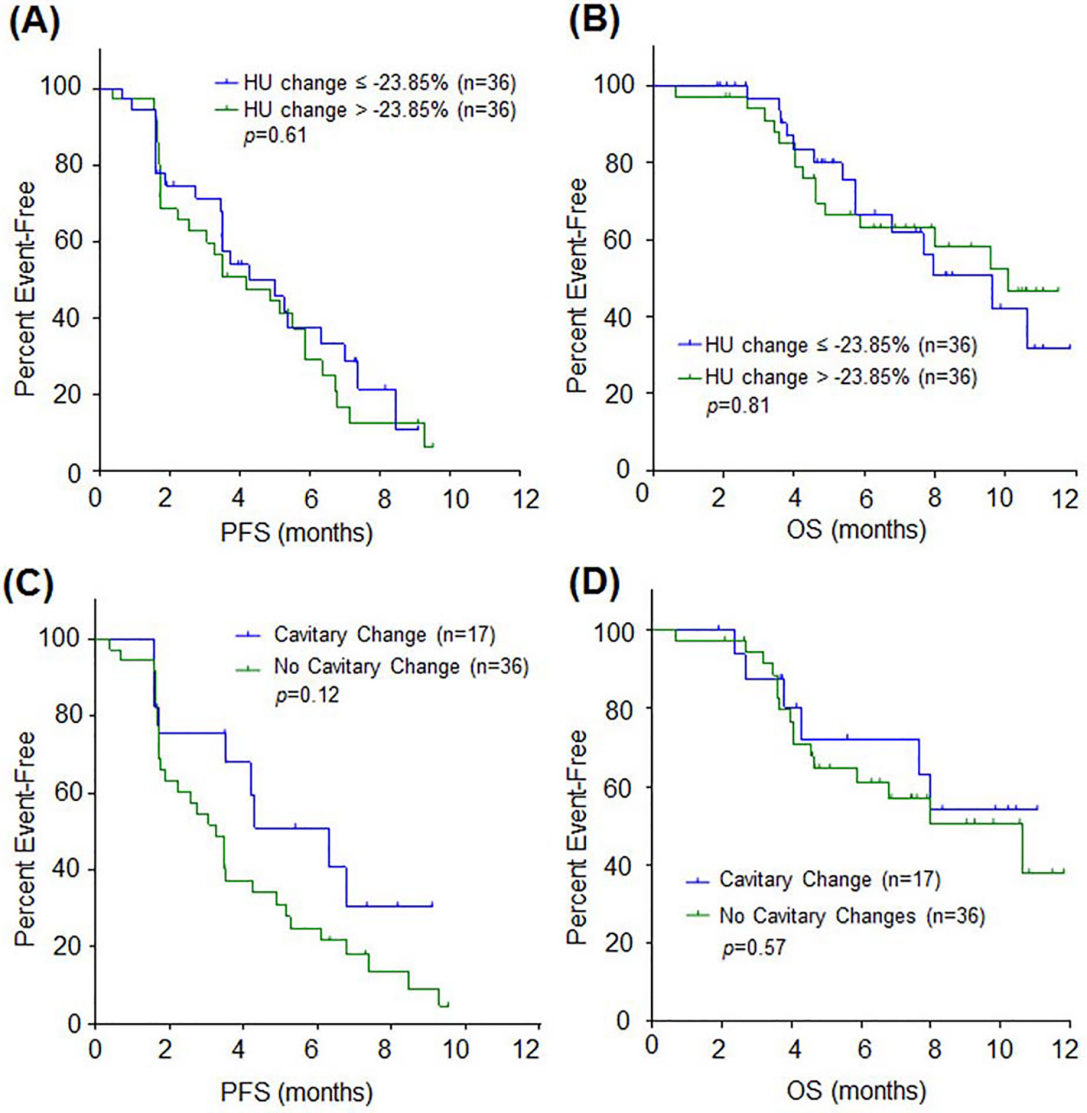
PFS and OS according to anti-angiogenic changes of regorafenib. (A-B) Kaplan-Meier curves of PFS (A) and OS (B) according to tumor attenuation change. (C-D) Kaplan-Meier curves of PFS (C) and OS (D) according to cavitary change of lung metastasis.

The magnitude of tumor attenuation decrease was not associated with treatment outcome. Using the median value of tumor attenuation change (-23.9%) as a cut-off, disease control rate (DCR) was 75.0% (27/36) in patients with higher tumor attenuation change and 69.4% (25/36) without higher change (*p* = 0.79). PFS and OS were also similar between the two groups (Fig 4A and 4B). No significant difference in treatment outcome was observed by using the HU cut-off (-30.52%) derived from mSUV change correlation analysis (data not shown).

Among the patients with lung metastasis, DCR was 82.4% (14/17) in patients showing cavitary changes and 63.9% (23/36) in patients without cavitary changes (*p* = 0.15). Development of cavitary change had no impact on PFS or OS (Fig 4C and 4D).

**Fig 5:**
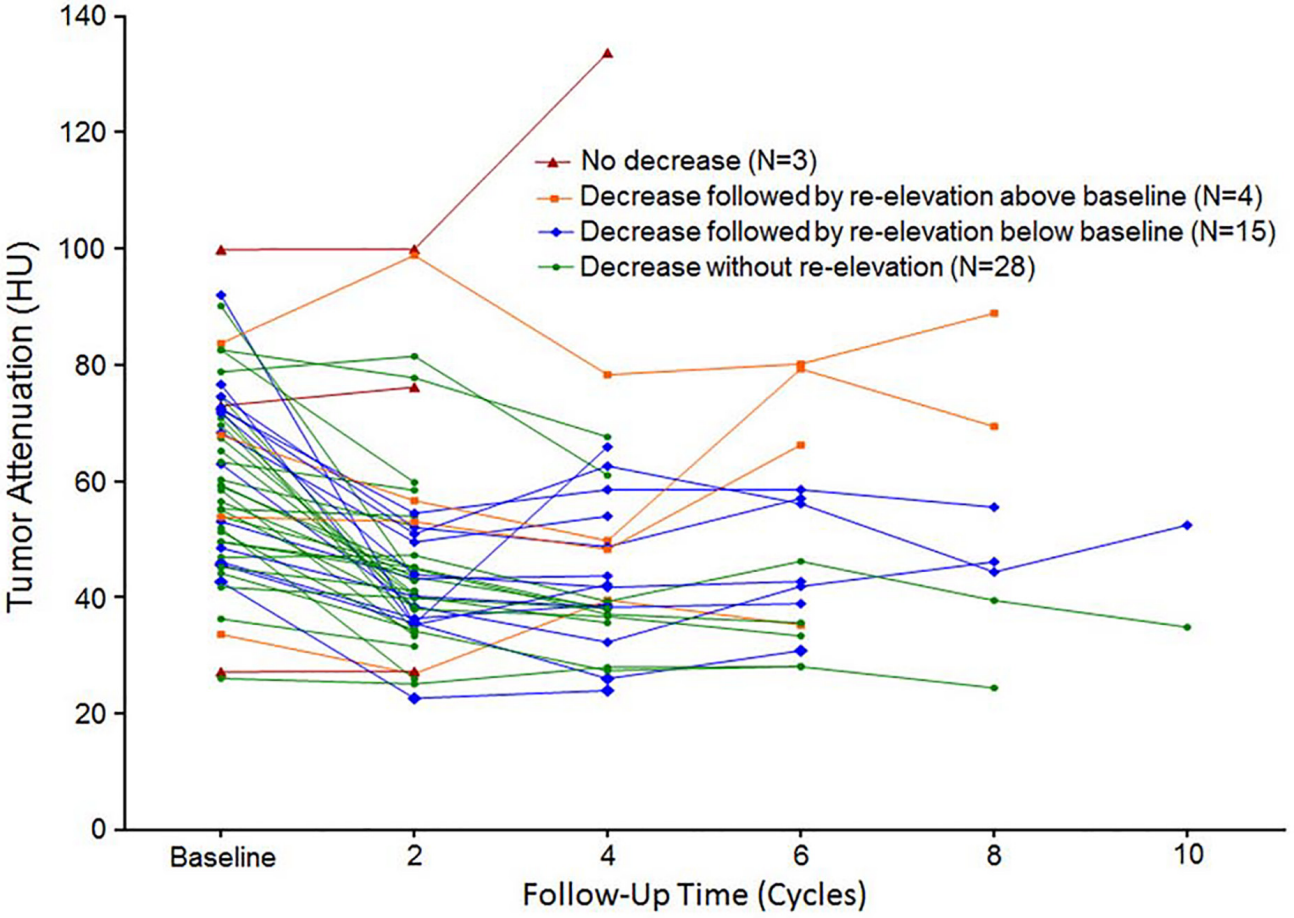
Tumor attenuation change at the time of PD by RECIST 1.1. Tumor attenuation until the time of PD is serially plotted in 50 patients who had PD according to RECIST 1.1. The decrease without re-elevation group includes 18 patients who had PD after 2 cycles but with decreased tumor attenuation and 10 patients with persistent decrease at the time of PD which occurred beyond 2 cycles.

At the time of PD according to RECIST 1.1, tumor attenuation was lower than baseline in 86.0% (43/50) of patients (Fig 5). In 18 patients, objective response was PD after two cycles but the tumor attenuation was decreased from baseline. In 10 patients, tumor attenuation was persistently decreased at the time of PD, which occurred beyond two cycles. 15 patients showed initial decrease in tumor attenuation followed by re-elevation but below baseline. In addition, cavitary change of lung metastasis persisted without refilling in 84.6% (11/13) of patients at the time of PD by RECIST 1.1.

## Discussion

In the present study, we show that radiological changes of regorafenib, namely decrease in tumor attenuation and cavitary change of lung metastasis, are frequently observed in metastatic colorectal cancer patients treated with regorafenib. These radiological changes most likely are the results of potent anti-angiogenic activity of regorafenib. Regorafenib can inhibit angiogenic kinases including VEGFR1, VEGFR2, VEGFR3, and TIE2 [1]. The anti-angiogenic activity of regorafenib is also demonstrated in preclinical animal models [1, 7–9]. The radiological changes analyzed herein have been reported in other cancers using different angiogenesis inhibitors [11–13]. Therefore, the radiological changes may be a good pharmacodynamic surrogate of anti-angiogenic activity.

Regorafenib effectively suppressed tumor vascularization evaluated by dynamic contrast-enhanced (DCE) magnetic resonance imaging (MRI) and microvessel area using immunostaining of endothelial marker CD31 in xenograft models [1, 7]. In another colon cancer xenograft model examining the effect of regorafenib, tumor perfusion and vascularity measured using DCE-CT showed good correlation with CD31 staining in tumor specimens [8]. Reduction in tumor perfusion assessed using DCE-MRI has been observed after regorafenib treatment in colorectal cancer patients in a phase 1 study [14]. In line with the study, we confirmed the radiological evidence of the anti-angiogenic activity of regorafenib in colorectal cancer patients. Tumor attenuation in contrast-enhanced CT decreased in the majority of the patients (87.5%) after two cycles of regorafenib treatment. The reduction of tumor attenuation was more frequently observed than the tumor size reduction (30.0%) suggesting that the anti-angiogenic activity may be a dominant mechanism of action of regorafenib in the clinical setting.

In the phase 3 studies of regorafenib in metastatic colorectal cancer, best overall response was SD in 41–45% of patients and only few patients (1–4%) showed PR [2, 4]. Hence, the main benefit of regorafenib in colorectal cancer may be from retardation of tumor growth rather than tumor shrinkage, which differs from cytotoxic chemotherapies. Considering the different mechanism of action of regorafenib, response evaluation and therapeutic decision solely based on size changes might be suboptimal and there is a need for alternative criteria. Similarly, in colorectal liver metastases treated with bevacizumab, there has been previous report demonstrating that morphologic changes evaluated by CT including attenuation pattern, may be more sensitive than RECIST 1.1 in predicting pathologic response [15]. In GIST patients treated with imatinib, Choi’s criteria, which measure both tumor size and density changes, can better predict treatment outcome [16]. However, the performance of Choi’s criteria was inferior to RECIST 1.1 for regorafenib in previously treated GIST, which suggests that optimal response criteria may vary according to the clinical settings [17]. Modified RECIST measuring arterial enhancing viable tumor was developed for hepatocellular carcinoma and this was useful in predicting efficacy of sorafenib [18, 19]. Alternative criteria incorporating tumor attenuation change in addition to size change have also been proposed for renal cell carcinoma patients receiving anti-angiogenic treatments [20, 21].

Tumor cavitation is frequently observed in non-small cell lung cancer patients treated with anti-angiogenic agents [12, 13, 22]. Although these studies have limitation of including patients treated with different anti-angiogenic agents or accompanying chemotherapy regimens, development of tumor cavitation was not associated with treatment outcome [12, 22]. As tumor cavitation may be an indicator of therapeutic activity of anti-angiogenic agents, alternate response evaluation criteria incorporating cavity diameter have been proposed [13]. Cavitation of lung metastasis have been reported in eight out of ten patients among a small subgroup of colorectal cancer patients treated with regorafenib in the phase 3 study [23]. In the present study, development of new cavity or increase in pre-existing cavity size of lung metastasis was observed in one third of the 53 patients with lung metastasis. The frequent occurrence of cavitary change in lung metastasis is another circumstantial evidence of potent clinical anti-angiogenic activity of regorafenib in colorectal cancer.

While the radiological changes representing anti-angiogenic activity were frequently observed, we could not find a correlation between the radiological changes and treatment outcome. There can be a number of speculative explanations for this unexpected finding. Although the radiological changes may be good pharmacodynamic surrogates of anti-angiogenic activity, suppression of anti-proliferative kinases could be more important in determining the treatment outcome. Biomarkers of anti-proliferative effect of regorafenib should be studied in future studies. In addition, we showed that tumor attenuation change has modest correlation with mSUV change after regorafenib treatment. However, the mechanism of mSUV decrease by regorafenib is unclear thus far and many factors including inhibition of angiogenesis, changes in glucose transport and tumor metabolism, and decreased tumor proliferation could be involved. We could not examine the association between mSUV decrease and treatment outcome as PET-CT evaluation was included in the later stage of the study and patients with the scans have short durations of follow-up.

Tumor size based treatment decision according to the RECIST 1.1 could also have contributed to the lack of association between radiological changes and treatment outcome. As shown in this study, there is no correlation between tumor size change and attenuation change. Moreover, decreased tumor attenuation at the time of PD according to RECIST 1.1 was observed in many patients. It is possible that these patients have discontinued regorafenib while still benefiting from its anti-angiogenic effect. Anti-angiogenic agents with cytostatic activities including regorafenib may need different treatment strategy. Continuous angiogenesis inhibition using bevacizumab beyond first progression has shown to improve survival in metastatic colorectal cancer.[24] Whether patients exhibiting persistent angiogenic suppression at the time of disease progression by RECIST might benefit from continuation of regorafenib should be studied in future clinical trials.

In summary, regorafenib showed prominent anti-angiogenic effect in colorectal cancer represented by decrease in tumor attenuation and cavitary change of lung metastasis. However, the anti-angiogenic effects were not associated with treatment outcome under the current conventional treatment paradigm. We may need to develop alternative evaluation and treatment strategies using regorafenib.

## Supporting Information

S1 File Lesional HU and mSUV. Lesional attenuation (HU) and mSUV data of the 64 matched lesions from 12 consecutive patients, at baseline and at follow-up imaging are given.
(XLSX)
